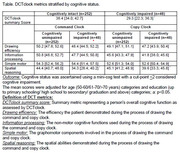# Digital Clock Assessment in South Asian Setting: Insights from the P‐CARRS study

**DOI:** 10.1002/alz.086976

**Published:** 2025-01-09

**Authors:** Ram Jagannathan, Deepa Mohan, Rani Komal, Dimple Kondal, Poongothai Subramani, Rima Pai, Mohammed K Ali, Sailesh Mohan, Suvarna Alladi, Nikhil Tandon, Dorairaj Prabhakaran, V Mohan, Allan I. Levey, Venkat Narayan KM, Felicia Goldstein

**Affiliations:** ^1^ Hubert Department of Global Health, Rollins School of Public Health, Atlanta, GA USA; ^2^ Madras Diabetes Research Foundation, Chennai, Tamil Nadu India; ^3^ Centre for Chronic Disease Control, New Delhi, Delhi India; ^4^ Hubert Department of Global Health, Rollins School of Public Healt, ATLANTA, GA USA; ^5^ National Institute of Mental Health and Neurosciences, Bangalore India; ^6^ All India Institute of Medical Sciences, New Delhi, Delhi India; ^7^ Emory University School of Medicine, Atlanta, GA USA; ^8^ Emory University, Atlanta, GA USA

## Abstract

**Background:**

Detection of presymptomatic individuals or those with subtle cognitive changes in midlife may prevent or slow the course of Alzheimer's Disease by identifying candidates for disease‐modifying treatments. Utilizing newer delivery approaches involving digital measures shows promise for cognitive phenotyping, early detection, ease of administration, and scoring, particularly in low‐resource settings. However, the feasibility of these approaches, along with their association with demographics and their effectiveness in detecting fine‐grained aspects of cognitive performance in low‐resource settings, remains unclear. We conducted a pilot study evaluating digital cognitive screening using an iPad‐administered clock drawing test in a diverse South Asian population residing in India.

**Methods:**

Individuals ≥ 50 years old (median: 61.0 years; range: 50‐89 years; female: 56%; education: up to primary schooling: 8%; high school to secondary: 65%; graduation and above: 27.4%) nested within the ongoing population‐based longitudinal Precision‐CARRS study representing socio‐demographically and linguistically diverse adults from Delhi and Chennai in India (n=300; 150/site) were studied. Participants were administered the FDA‐approved, iPad‐based clock drawing test using a commercially available Linus Health Digital Clock (DCTclock) screening tool. The DCTclock summary score, ranging from 0 to 100, was derived through AI analytics and provided various features, including drawing efficiency, information processing, simple motor skills, and visuospatial reasoning. Performance on the traditionally administered paper/pencil version of the Mini‐Cog test incorporating clock drawing and three‐item recall was used to classify participants as cognitively impaired (0‐2 points) or intact (3‐5 points).

**Results:**

DCTclock was administered in ∼3‐4 minutes, and only 0.7% of clocks were unanalyzable due to missing components. Older age and lower education (p<0.0001) were associated with worse DCTclock summary scores. There were no sex differences in performance (p=0.90). Compared to those cognitively intact based on the Mini‐Cog scores (n=252), the impaired group (n=48) performed significantly worse on the DCTclock summary score and spatial reasoning components (Table).

**Conclusion:**

Screening for cognitive status using digital clock measurement is highly feasible and takes a short amount of time to administer in low‐resource settings. The DCTclock provides fine‐grained measures of performance not available on traditional paper/pencil measures, which may prove sensitive to early detection of cognitive impairment.